# Stability Analysis of Multi-Sensor Kalman Filtering over Lossy Networks

**DOI:** 10.3390/s16040566

**Published:** 2016-04-20

**Authors:** Shouwan Gao, Pengpeng Chen, Dan Huang, Qiang Niu

**Affiliations:** 1 Key Laboratory of Gas and Fire Control for Coal Mines, China University of Mining and Technology, Xuzhou 221116, China; gaoshouwan@cumt.edu.cn; 2 School of Computer Science and Technology, China University of Mining and Technology, Xuzhou 221116, China; hdts15170041p2@cumt.edu.cn (D.H.); niuq@cumt.edu.cn (Q.N.)

**Keywords:** Kalman filtering, packet losses, distributed sensing, stability analysis, Markov process

## Abstract

This paper studies the remote Kalman filtering problem for a distributed system setting with multiple sensors that are located at different physical locations. Each sensor encapsulates its own measurement data into one single packet and transmits the packet to the remote filter via a lossy distinct channel. For each communication channel, a time-homogeneous Markov chain is used to model the normal operating condition of packet delivery and losses. Based on the Markov model, a necessary and sufficient condition is obtained, which can guarantee the stability of the mean estimation error covariance. Especially, the stability condition is explicitly expressed as a simple inequality whose parameters are the spectral radius of the system state matrix and transition probabilities of the Markov chains. In contrast to the existing related results, our method imposes less restrictive conditions on systems. Finally, the results are illustrated by simulation examples.

## 1. Introduction

**Figure 1:**
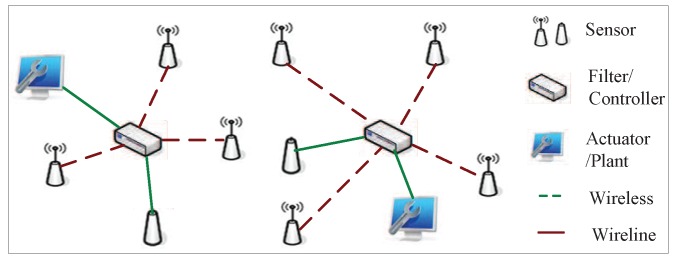
Distributed systems.

The Kalman filter and its variations have great potential in many applications involving detection, tracking and control [1,2,3]. Recently, the problem of filtering for distributed systems has attracted increasing attention due to the advantages, such as low cost, reduced weight and inherent robustness. As shown in Figure 1, in such distributed systems, sensor measurements and final signal processing usually take place at different physical locations and, thus, require a wireless or wireline communication network to exchange information. In contrast to traditional filtering problems, one main issue of these systems is that packet losses are unavoidable because of congestion and transmission errors in communication channels. How missing data affect the performance of filtering schemes is of significant interest.

Similar to filtering with network packet losses, early work has studied the problem of estimation with missing data at certain time points, where the observation may drop the signal that has errors, *i.e.*, such an observation may contain noise alone. By modeling the uncertainty in observation processes as an independent and identically distributed (i.i.d.) binary random variable sequence, the author in [4] proposes minimum mean-square error (MMSE) estimators, which are of a recursive form and similar to the Kalman filter. In [5], the authors generalize the work of [4] by investigating the filtering problem under the assumption that the uncertainty is not necessarily i.i.d. Moreover, when the data missing process can be modeled as an i.i.d. sequence with a known probability of the occurrence of signal losses, the work [6] derives sufficient conditions for the uniform asymptotic stability of the MMSE filter. It is noted that in the scenario of [6], since the error covariance is governed by a deterministic equation, the stability results can be obtained by defining an equivalent system where the observations contain the signal with probability one.

In the recent research for network models, a large number of works has been reported on the stability analysis of Kalman filtering (see, e.g., [7,8,9,10,11,12,13,14,15,16] and the references therein), and most of them account for possible observation losses. In the aforementioned literature, there have been basically two different methods to model the packet loss process in network systems. An arguably popular method is to describe the packet loss process as an i.i.d. Bernoulli sequence [17]. Recently, some results have been published on such a model; see, e.g., [9,10,11]. The second method is to employ the Markov process to model the packet loss phenomenon [18]. Such a model has been adopted in [12,13,14,15,16] to deal with the filtering problem with packet losses. Though under the i.i.d. model, filtering stability may usually be effectively analyzed by solving a modified Riccati recursion, an i.i.d. process is inadequate to describe the states of network channels that do not vary independently in time. Hence, compared to the i.i.d. model, a remarkable advantage of a Markovian packet loss model is that it can capture the possible temporal correlation of network channels.

It should be pointed out that almost all of the aforementioned results are obtained under the single sensor assumption. Note that from the view of state estimation, the single sensor case also includes the multi-sensor condition, where all of the measurements from different sensors can be encapsulated together and sent to the remote filter by a common channel. Under the single sensor assumption, the filter either receives the observations in full or losses them completely. Obviously, such an assumption may not hold in many practical distributed filtering systems. In these systems, the sensors are usually placed in a wide area; the measurements coming from different sensors cannot be encoded together; and they must be sent over multiple different channels. In contrast to the case of a single sensor, the main difficulty induced by multiple sensors is that observations may be partially lost. For the partial packet loss case, the explicit characterization of stability conditions for the Kalman filter is extremely challenging. Fortunately, by modeling the packet loss processes as i.i.d. Bernoulli sequences, the authors in [19] obtain a sharp transition curve, which is the function of loss rates and can separate the stable and unstable regions of the error covariance matrix, for a couple of special systems, including cases in which the state matrix of the system has a single unstable mode and all observation matrices are invertible. However, they cannot explicitly find the sharp transition curve for other systems, except providing a lower bound and an upper bound for this sharp transition curve. Moreover, when the data dropout process is Markovian, the work [20] offers the sufficient condition for the covariance stability. Nevertheless, this work fails to offer necessary and sufficient conditions. In addition, the restrictive condition in [20] is very strong, that is the observation matrix is invertible. More recently, for the scenario of multiple sensors, the authors in [21] derive necessary and sufficient stability conditions for the estimation error covariance. However, they are unable to characterize stability conditions as a simple form of inequalities, except for the second-order systems with the i.i.d. model. So far, few results in the existing literature are concerned with the explicit filtering stability conditions for multi-sensor systems with partial Markovian packet losses. This is the motivation of the present paper.

Our work studies the stability of Kalman filter under the multi-sensor case. We extend the results in [12] by allowing the measurements coming from different sensors to be sent by multiple channels. Under our scenario, the whole observations consist of multiple packets coming from corresponding sensors, and part or all of the observations may be lost. For the sake of simplicity, we first assume that the system is set with two sensors and give necessary and sufficient stability conditions for the error covariance matrix. Then, we extend our results to a more general case where the multi-sensor scenario is considered. Different sensors are possibly subject to different packet losses. It is worth pointing out that, similar to [12], this work gives the stability criterion in the form of a simple inequality whose parameters are the spectral radius of the system state matrix and the transition probabilities of the Markovian packet loss processes. Thus, based on our stability criterion, one may understand how the packet losses affect the stability.

The remainder of the paper is organized as follows. Section 2 formulates the problem under consideration. In Section 3, a necessary condition for stability is derived under standard assumptions, and the necessary condition is shown to be also sufficient for certain classes of systems. Differences between our results and previous ones are discussed in Section 4. Section 5 presents simulation examples, and some concluding remarks are drawn in Section 6.

Notation: throughout this paper, a symmetric matrix P>0 $\left(P\geq 0\right)$ denotes that *P* is a positive definite (semi-definite) matrix, and the relationship A>B $\left(A\geq B\right)$ means A−B>0 $\left(A-B\geq 0\right)$. Matrix *I* represents the identity matrix with a compatible dimension. N, R and C are used to mean the sets of nonnegative integers, real numbers and complex numbers, respectively. $\text{Tr}\left(\cdot \right)$ denotes the matrix trace, and $\rho \left(\cdot \right)$ represents the spectral radius of a matrix.

## 2. Problem Formulation

Consider the following linear discrete-time stochastic system:

(1) $$x_{k+1}=Ax_{k}+w_{k}$$

(2) $$y_{k}=Cx_{k}+v_{k}$$

where $x_{k}\in R^{n}$ is the system state and $y_{k}\in R^{m}$ is the measured output. *A* and *C* are constant matrices of compatible dimensions, and *C* is of full row rank. Both $w_{k}\in R^{n}$ and $v_{k}\in R^{m}$ are white Gaussian noises with zero means and covariance matrices Q>0 and R>0, respectively. Assume that the initial state $x_{0}$ is also a random Gaussian vector of mean ${\bar{x}}_{0}$ and covariance $P_{0}>0$. Moreover, $w_{k}$, $v_{k}$ and $x_{0}$ are mutually independent.

**Figure 2:**
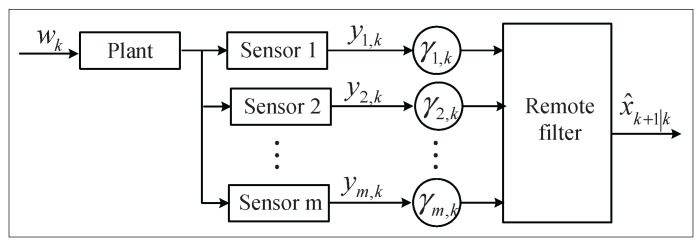
Diagram of a networked filtering system under distributed sensing.

The estimation problem under consideration is illustrated in Figure 2. For simplicity, we first assume the networked system is set with two sensors. Note that all of our results extend to the more sensors case easily. Under the two-sensor assumption, the observation $y_{k}$ consists of two parts $y_{1,\;k}$ and $y_{2,\;k}$, which are transmitted through different channels. Then, Equation (2) can be rewritten as follows:

(3) $$\left[\begin{array}{c} y_{1,\;k}\\ y_{2,\;k} \end{array}\right]=\left[\begin{array}{c} C_{1}\\ C_{2} \end{array}\right]x_{k}+\left[\begin{array}{c} v_{1,\;k}\\ v_{2,\;k} \end{array}\right]$$

where $y_{1,\;k},\;v_{1,\;k}\in R^{m_{1}}$ and $y_{2,\;k},\;v_{2,\;k}\in R^{m_{2}}$. The covariance matrices of $v_{1,\;k}$ and $v_{2,\;k}$ are $R_{11}>0$ and $R_{22}>0$, respectively. Comparing to Equation (2), it is obvious that: $y_{k}=\left[\begin{array}{c} y_{1,\;k}\\ y_{2,\;k} \end{array}\right]$, $C=\left[\begin{array}{c} C_{1}\\ C_{2} \end{array}\right]$ , and $R=\left[\begin{array}{cc} R_{11} & R_{12}\\ R_{21} & R_{22} \end{array}\right]$. Under our scenario, the sensor measurements $y_{1,\;k}$ and $y_{2,\;k}$ are transmitted to the remote filter via different unreliable communication channels. Due to fading and/or congestion, the communication channels may be subject to random packet losses. Two time-homogeneous binary Markov chains $\gamma_{1,\;k}$ and $\gamma_{2,\;k}$ are adopted to describe, respectively, the packet loss processes in the two channels. Note that such a Markov model is more general and realistic than the i.i.d. case studied in [19], since the Markov process can capture the temporal correlation of the channel variation. We assume that $y_{i,\;k}$, $i\in \left\{1,\;2\right\}$, is received correctly in time step *k* if $\gamma_{i,\;k}=1$, while there is a packet loss if $\gamma_{i,\;k}=0$. In addition, we denote the transition probability matrix of $\gamma_{i,\;k}$ as follows:

(4) $$\begin{array}{c} \Theta_{i}^{+}={\left(P\left\{\gamma_{i,\;k+1}=j\left|\gamma_{i,\;k}=l\right.\right\}\right)}_{j,\;l\in \left\{0,\;1\right\}}=\left[\begin{array}{cc} 1-q_{i} & q_{i}\\ p_{i} & 1-p_{i} \end{array}\right],\;i\in \left\{1,\;2\right\} \end{array}$$

where $q_{i}$ and $p_{i}$, respectively, are the recovery rate and failure rate of the i-th channel. It is further assumed that $0<p_{i},\;q_{i}<1$, so that the Markov chain ${\left\{\gamma_{i,\;k}\right\}}_{k\in N}$ is ergodic. The distributions of the process $\left\{\gamma_{i,\;k}\right\}$ are determined by the corresponding channel gains, bit-rates, power levels and temporal correlation. It is obvious that a smaller value of $p_{i}$ and a larger value of $q_{i}$ indicate that the i-th channel is more reliable. To avoid any trivial case, we assume that $\gamma_{1,\;k}$ and $\gamma_{2,\;k^{\prime }}$ are independent for every *k* and $k^{\prime }$.

Introduce $S_{k}$ to indicate the packet loss status in the whole network at time step *k*. In view of the above analysis and assumption, we easily obtain that the process ${\left\{S_{k}\right\}}_{k\in N}$ is a four-state Markov chain. To be more specific, the following indicator function is given:

(5) $$S_{k}=\left\{\begin{array}{c} \varsigma_{00},\;\;\gamma_{1,\;k}=0\;\;\text{and}\;\;\gamma_{2,\;k}=0\\ \varsigma_{01},\;\;\gamma_{1,\;k}=0\;\;\text{and}\;\;\gamma_{2,\;k}=1\\ \varsigma_{10},\;\;\gamma_{1,\;k}=1\;\;\text{and}\;\;\gamma_{2,\;k}=0\\ \varsigma_{11},\;\;\gamma_{1,\;k}=1\;\;\text{and}\;\;\gamma_{2,\;k}=1 \end{array}\right.$$

Then, it follows from Equations (4) and (5) that the Markov process ${\left\{S_{k}\right\}}_{k\in N}$ has a transition probability matrix given by:

(6) $$\Psi^{+}=\left[\begin{array}{cccc} \left(1-q_{1}\right)\left(1-q_{2}\right) & \left(1-q_{1}\right)q_{2} & q_{1}\left(1-q_{2}\right) & q_{1}q_{2}\\ \left(1-q_{1}\right)p_{2} & \left(1-q_{1}\right)\left(1-p_{2}\right) & q_{1}p_{2} & q_{1}\left(1-p_{2}\right)\\ p_{1}\left(1-q_{2}\right) & p_{1}q_{2} & \left(1-p_{1}\right)\left(1-q_{2}\right) & \left(1-p_{1}\right)q_{2}\\ p_{1}p_{2} & p_{1}\left(1-p_{2}\right) & \left(1-p_{1}\right)p_{2} & \left(1-p_{1}\right)\left(1-p_{2}\right) \end{array}\right]$$

In order to simplify the analysis, we classify the packet loss status of networks into two categories by the same method as that in [20]:

(7) $$S_{k}=\left\{\begin{array}{c} \varsigma_{L},\;\;\gamma_{1,\;k}=0\;\;\text{and}\;\;\gamma_{2,\;k}=0\;\;\;\;\\ \varsigma_{R},\;\;\gamma_{1,\;k}=1\;\;\text{or/and}\;\;\gamma_{2,\;k}=1 \end{array}\right.$$

Associated with Equation (7), we, thus, model the packet loss status $S_{k}$ as a two-state Markov process with transition probability matrix:

(8) $$\Pi^{+}={\left(P\left\{S_{k+1}=\varsigma_{j}\left|S_{k}=\varsigma_{l}\right.\right\}\right)}_{\varsigma_{j},\;\varsigma_{l}\in \Omega }=\left[\begin{array}{cc} 1-\eta & \eta \\ \mu & 1-\mu \end{array}\right]$$

where $\Omega =\left\{\varsigma_{L},\;\varsigma_{R}\right\}$ is the state space of the Markov chain. Note that with Equation (6), we further obtain that 0<η,μ<1, which ensures the ergodic property of the above two-state Markov process. Specifically, one can easily derive the following equation:

(9) $$1-\eta =\left(1-q_{1}\right)\left(1-q_{2}\right)$$

**Remark 1.**  The problem of mean square stability for Kalman filtering with Markovian packet losses has been studied in [12], where the single sensor case is considered. Our paper generalizes [12] by allowing partial observation losses. In our scenario, the measurements coming from different sensors can be transmitted via different communication channels. Obviously, our results are more meaningful and general in practical applications, since the adopted model can be easily adjusted to describe the single sensor situation.

Based on the history $Z_{k}={\left[\begin{array}{c} y_{0},\;\cdots \;,\;y_{k} \end{array}\right]}^{H}$ and $Y_{k}={\left[\begin{array}{c} \gamma_{0},\;\cdots \;,\;\gamma_{k} \end{array}\right]}^{H}$, where $\gamma_{k}=\left\{\begin{array}{c} \gamma_{1,\;k};\;\gamma_{2,\;k} \end{array}\right\}$ and $A^{H}$ is the conjugate transpose of *A*, one can define the filtering and one-step prediction equations corresponding to the optimal estimation as follows:

(10) $${\hat{x}}_{k|k}=E\left[\begin{array}{c} x_{k}|Z_{k},\;Y_{k} \end{array}\right]$$

(11) $${\hat{x}}_{k+1|k}=E\left[\begin{array}{c} x_{k+1}|Z_{k},\;Y_{k} \end{array}\right]$$

The associated estimation and prediction error covariance matrices can be written by:

(12) $$P_{k|k}=E\left[\begin{array}{c} \left(\begin{array}{c} x_{k}-{\hat{x}}_{k|k} \end{array}\right){\left(\begin{array}{c} x_{k}-{\hat{x}}_{k|k} \end{array}\right)}^{H}|Z_{k},\;Y_{k} \end{array}\right]$$

(13) $$P_{k+1|k}=E\left[\begin{array}{c} \left(\begin{array}{c} x_{k+1}-{\hat{x}}_{k+1|k} \end{array}\right){\left(\begin{array}{c} x_{k+1}-{\hat{x}}_{k+1|k} \end{array}\right)}^{H}|Z_{k},\;Y_{k} \end{array}\right]$$

Our approach is to analyze the estimation error of ${\hat{x}}_{k+1|k}$; hence, the details for the recursion of ${\hat{x}}_{k|k}$ and ${\hat{x}}_{k+1|k}$ are omitted here. Considering [19], we have the following recursions for estimation error covariance matrices:

(14) $$\begin{array}{cc} P_{k|k} & =P_{k|k-1}-\gamma_{1,\;k}\gamma_{2,\;k}P_{k|k-1}C^{H}{\left(\begin{array}{c} CP_{k|k-1}C^{H}+R \end{array}\right)}^{-1}CP_{k|k-1}\\ & -\gamma_{1,\;k}\left(\begin{array}{c} 1-\gamma_{2,\;k} \end{array}\right)P_{k|k-1}{C_{1}}^{H}{\left(\begin{array}{c} C_{1}P_{k|k-1}{C_{1}}^{H}+R_{11} \end{array}\right)}^{-1}C_{1}P_{k|k-1}\\ & -\gamma_{2,\;k}\left(\begin{array}{c} 1-\gamma_{1,\;k} \end{array}\right)P_{k|k-1}{C_{2}}^{H}{\left(\begin{array}{c} C_{2}P_{k|k-1}{C_{2}}^{H}+R_{22} \end{array}\right)}^{-1}C_{2}P_{k|k-1} \end{array}$$

(15) $$P_{k+1|k}=AP_{k|k}A^{H}+Q$$

In addition, the initial value is $P_{0|-1}=P_{0}$. For simplicity of exposition, we slightly abuse the notation by substituting $P_{k+1|k}$ with $P_{k+1}$. To analyze the statistical properties of the estimation error covariance matrix $P_{k}$, we recall the following definition from [12].

**Definition 1.**  The sequence ${\left\{P_{k}\right\}}_{k\in N}$ is said to be stable if $\sup_{k\in N}E\left[P_{k}\right]<\infty$ for any $P_{0}>0$, where the expectation is taken on ${\left\{\gamma_{k}\right\}}_{k\in N}$ with the initial value $\gamma_{0}$ being any Bernoulli random variable.

Here, $E\left[P_{k}\right]$ denotes the mean of prediction error covariance at time step *k*. Our objective is to derive necessary and sufficient conditions for the stability of ${\left\{P_{k}\right\}}_{k\in N}$ under the multi-sensor environment with partial observation losses. It should be pointed out that, for the Kalman filter with partial Markovian packet losses, the stability has been studied in [20], while their approach imposes more restriction on systems such that the results are conservative. In this paper, we propose a completely different method to obtain the main results. To make the model nontrivial, we make the following assumptions throughout the paper.

**Assumption 1.**  All of the eigenvalues of A lie outside the unit circle.

**Assumption 2.**  The system $\left(C,\;A\right)$ is observable.

**Assumption 3.**  $P_{0}$, Q and R are all identity matrices with appropriate dimensions.

## 3. Stability Conditions for Error Covariance

In the following, we will derive our stability conditions for the estimation error covariance matrix. We first give the necessary condition for stability under Assumptions 1–3. Then, we prove that the necessary condition is also sufficient for a certain class of systems.

### 3.1. Necessary Condition for Covariance Stability

In order to facilitate our presentation, we denote $E^{\varsigma_{i}}\left[\cdot \right]$ as the mathematical expectation operator conditioned on the initial state $S_{0}=\varsigma_{i}$, where $\varsigma_{i}\subset \Omega$. The following lemma will be used in the proof of necessary condition for stability.

**Lemma 1.**  The matrix inequality $\sup_{k\in N}E\left[P_{k}\right]<\infty$ holds if and only if $\sup_{k\in N}E^{\varsigma_{L}}\left[P_{k}\right]<\infty$ and $\sup_{k\in N}E^{\varsigma_{R}}\left[P_{k}\right]<\infty$.

**Proof.**  By revising Lemma 2 in [12], we can easily get the proof; hence, the details are omitted. ☐

**Theorem 2.**  If System (1) and (3) satisfies Assumptions 1–3 and the packet loss process is described by a time-homogeneous Markov chain with transition probability matrix (8), a necessary condition for $\sup_{k\in N}E\left[P_{k}\right]<\infty$ is $\rho {\left(A\right)}^{2}\left(1-q_{1}\right)\left(1-q_{2}\right)<1$.

**Proof.**  To simplify the notation, we define $\pi_{j}^{\varsigma_{i}}=P\left\{S_{j}=\varsigma_{i}\right\}$, $\varsigma_{i}\in \Omega$ and $\pi_{j}=\left[\begin{array}{c} \pi_{j}^{\varsigma_{L}},\;\pi_{j}^{\varsigma_{R}} \end{array}\right]$. It immediately follows from Equation (8) that $\pi_{j+1}=\pi_{j}\Pi^{+}$. Jointly with 0<η,μ<1, one has $\pi_{j}^{\varsigma_{i}}>0$ for any j≥1. In addition, we obtain that the two-state Markov chain ${\left\{S_{k}\right\}}_{k\in N}$ has a unique stationary distribution $\pi =\left[\begin{array}{c} \pi^{\varsigma_{L}},\;\pi^{\varsigma_{R}} \end{array}\right]$, specifically, $\pi^{\varsigma_{L}}=\frac{\mu }{\eta +\mu }$ and $\pi^{\varsigma_{R}}=\frac{\eta }{\eta +\mu }$. By letting $\pi_{-}^{\varsigma_{L}}=\inf_{j\geq 1}\pi_{j}^{\varsigma_{L}}$, the following inequality holds for any j≥2,

(16) $$\prod_{i=j}^{k}P\left\{S_{i}=\varsigma_{L}\left|S_{j-1}=\varsigma_{L}\right.\right\}P\left\{S_{j-1}=\varsigma_{L}\right\}\geq \pi_{-}^{\varsigma_{L}}{\left(1-\eta \right)}^{k-j+1}$$

In view of Equation (7), it is clear that the above inequality implies that:

(17) $$\begin{array}{cc} E & \left[\prod_{i=j}^{k}\left(1+\gamma_{1,\;i}\gamma_{2,\;i}-\gamma_{1,\;i}-\gamma_{2,\;i}\right)\right]\\ & \geq E\left[\prod_{i=j}^{k}\left(1+\gamma_{1,\;i}\gamma_{2,\;i}-\gamma_{1,\;i}-\gamma_{2,,\;i}\right)\left|\gamma_{1,\;j-1}=\gamma_{2,\;j-1}=0\right.\right]P\left\{\gamma_{1,\;j-1}=0,\;\gamma_{2,\;j-1}=0\right\}\\ & \geq \pi_{-}^{\varsigma_{L}}{\left(1-\eta \right)}^{k-j+1} \end{array}$$

On the other hand, considering Assumption 3 and a similar argument as that used in Theorem 4 of [12], we obtain for any k≥2:

(18) $$\begin{array}{cc} P_{k+1} & \geq \left(1+\gamma_{1,\;k}\gamma_{2,\;k}-\gamma_{1,\;k}-\gamma_{2,\;k}\right)AP_{k}A^{H}+Q\\ & \geq \sum_{j=1}^{k}\left(\underset{i=j}{\prod^{k}}\;\left(1+\gamma_{1,\;i}\gamma_{2,\;i}-\gamma_{1,\;i}-\gamma_{2,\;i}\right)\right)A^{k-j+1}{\left(A^{k-j+1}\right)}^{H} \end{array}$$

Take expectation with respect to $\gamma_{1,\;i}$ and $\gamma_{2,\;i}$ on both sides of Inequality (18), and note Inequality (17); one can prove:

(19) $$E\left[P_{k+1}\right]\geq \pi_{-}^{\varsigma_{L}}\sum_{j=1}^{k-1}{\left(1-\eta \right)}^{j}A^{j}{\left(A^{j}\right)}^{H}$$

In the following, we show bounded conditions for the right side of Inequality (19) when k→∞. To prove this, we introduce a nonsingular matrix *U*, such that AU=UJ, where *J* is the Jordan canonical form of *A* with the form $J=\text{diag}\left\{\begin{array}{c} J_{1},\;\cdots \;,\;J_{d} \end{array}\right\}\in C^{n\times n}$, and $J_{i}$ corresponds to the eigenvalue $\lambda_{i}$. Letting $\lambda_{\min}^{{}^{\prime }}$ be the smallest eigenvalue of $U^{-1}U^{-H}$, one can obtain $\lambda_{\min}^{{}^{\prime }}>0$. Together with Inequality (19), it is easy to verify:

(20) $$\sum_{j=1}^{k-1}{\left(1-\eta \right)}^{j}A^{j}{\left(A^{j}\right)}^{H}\geq \lambda_{\min}^{{}^{\prime }}U\sum_{j=1}^{k-1}{\left(1-\eta \right)}^{j}J^{j}{\left(J^{j}\right)}^{H}U^{H}$$

Then, by use of Lemma 1, it can be checked that $\sup_{k\in N}E\left[P_{k}\right]<\infty$ implies:

(21) $$\sup_{k\in N}E^{\varsigma_{L}}\left[\sum_{j=1}^{k-1}{\left(1-\eta \right)}^{j}J^{j}{\left(J^{j}\right)}^{H}\right]<\infty$$

Along the same line of the proof of Theorem 3 in [12], we conclude that a necessary condition for (21) is ${\left|\lambda_{i}\right|}^{2}\left(1-\eta \right)<1$. Note that $\lambda_{i}$ is an arbitrary eigenvalue of *A*; now, it is clear that $\rho {\left(A\right)}^{2}\left(1-q_{1}\right)\left(1-q_{2}\right)<1$. This completes the proof. ☐

### 3.2. Stability for Non-Degenerate Systems

In this subsection, we show that the condition given by Theorem 2 is also sufficient under the assumption that $\left(C,\;\;A\right)$ is a non-degenerate pair. To prove this, we adopt the following definitions introduced in [9].

**Definition 2.**  Assume the system $\left(C,\;A\right)$ has the form of $A=diag\left(\lambda_{1},\;\cdots \;,\;\lambda_{n}\right)$ and $C=\left[C_{1},\;\cdots \;,\;C_{n}\right]$. A block of the system is defined as a subsystem corresponding to $\left(\begin{array}{c} A_{L}=diag\left(\lambda_{i_{1}},\;\cdots \;,\;\lambda_{i_{l}}\right),\;C_{L}=\left[C_{i_{1}},\;,\cdots \;,\;C_{i_{l}}\right] \end{array}\right)$, where $L=\left\{l_{1},\;\cdots \;,\;l_{n}\right\}\subset \left\{1,\;\cdots \;,\;n\right\}$. As a special case, a block satisfying $\left|\lambda_{i_{1}}\right|=\cdots =\left|\lambda_{i_{l}}\right|$ is called an equi-block.

**Definition 3.**  If C is of full column rank, we say the system $\left(C,\;A\right)$ is one-step observable.

**Definition 4.**  If an equi-block is one-step observable, it is non-degenerate. Moreover, if all equi-blocks of the system are non-degenerate, the system is non-degenerate. Otherwise, it is degenerate.

It is obvious that the non-degenerate assumption is stronger than the observable condition, but weaker than the one-step observable case. Before we continue on, let us define:

(22) $$\Lambda_{k}=\sum_{i=1}^{k+1}A^{-iH}C^{H}M_{k+1-i}CA^{-i}+A^{-\left(k+1\right)H}A^{-\left(k+1\right)}$$

(23) $$\Xi_{k}=\sum_{i=1}^{k+1}A^{-iH}C^{H}M_{i}CA^{-i}+A^{-\left(k+1\right)H}A^{-\left(k+1\right)}$$

(24) $$\Gamma =\sum_{i=1}^{\infty }A^{-iH}C^{H}M_{i}CA^{-i}$$

where $M_{i}=\text{diag}\left(\gamma_{1,\;i}I_{m_{1}},\;\gamma_{2,\;i}I_{m_{2}}\right)$. Then, to obtain the main results, we give the following lemma by recalling [9].

**Lemma 3.**  *Under Assumptions 1–3, the prediction error covariance matrix $P_{k}\;\left(k\in N\right)$ is bounded by:*

(25) $$\alpha_{1}\Lambda_{k}^{-1}\leq P_{k+1}\leq \beta_{1}\Lambda_{k}^{-1}$$

*where $\alpha_{1}$ and $\beta_{1}$ are strictly positive constant numbers.*

It follows from Equation (6) that Γ is invertible almost everywhere. Thus, there is no loss of generality to assume that the inverse of Γ is well defined in the rest of the paper. Similar to [9], we see that the matrix Γ plays an important role in proving the stability of ${\left\{P_{k}\right\}}_{k\in N}$, since it bounds $E\left[P_{k}\right]$ as in the following theorem.

**Theorem 4.**  *Assume that System (1) and (3) satisfies Assumptions 1–3 and that the packet loss process is described by a time-homogeneous Markov chain with transition probability matrix (8). Then, a necessary and sufficient condition for $\sup_{k\in N}E\left[P_{k}\right]<\infty$ is $E\left[\Gamma^{-1}\right]<\infty$. Moreover, there exist strictly positive constant numbers $\alpha_{2}$ and $\beta_{2}$, such that:*

(26) $$\alpha_{2}E\left[\Gamma^{-1}\right]\leq \sup_{k\in N}E\left[P_{k}\right]\leq \beta_{2}E\left[\Gamma^{-1}\right]$$

**Proof.**  Proof of the right-hand side: It follows readily from Equation (4) that the Markov chain ${\left\{\gamma_{i,\;k}\right\}}_{k\in N}$, $i\in \left\{1,\;2\right\}$, has a unique stationary distribution. Assume that ${\left\{\gamma_{i,\;k}\right\}}_{k\in N}$ starts at its stationary distribution, then $\left\{\gamma_{i,\;k}\right\}$ has the same distribution for any ${}_{k\in N}$. Moreover, under the assumption, it is easy to check:

(27) $$\Theta_{i}^{-}={\left(P\left\{\gamma_{i,\;k}=j\left|\gamma_{i,\;k+1}=l\right.\right\}\right)}_{j,\;l\in \left\{0,\;1\right\}}=\left[\begin{array}{cc} 1-q_{i} & q_{i}\\ p_{i} & 1-p_{i} \end{array}\right],\;\;i\in \left\{1,\;2\right\}$$

According to the similar idea in [12], we introduce a measurable function $f:\;R^{m\times m}\to R^{n\times n}$. Thus, one has:

$$\begin{array}{cc} E & \left[f\left(M_{k},\;,\cdots \;,\;M_{0}\right)\right]\\ & \overset{\left(a\right)}{=}\;\sum_{\begin{array}{c} i_{1,\;j},i_{2,\;j}\in \left\{0,\;1\right\},\\ 0\leq j\leq k \end{array}}E\left[f\left(\text{diag}\left(i_{1,\;k}I_{m_{1}},\;i_{2,\;k}I_{m_{2}}\right),\;\cdots \;,\;\text{diag}\left(i_{1,\;0}I_{m_{1}},\;i_{2,\;0}I_{m_{2}}\right)\right)\right]\\ & \;\;\;\times P\left\{\gamma_{1,\;k}=i_{1,\;k},\;\gamma_{2,\;k}=i_{2,\;k},\;\cdots \;,\;\gamma_{1,\;0}=i_{1,\;0},\;\gamma_{2,\;0}=i_{2,\;0}\right\}\\ & \overset{\left(b\right)}{=}\;\sum_{\begin{array}{c} i_{1,\;j},i_{2,\;j}\in \left\{0,\;1\right\},\\ 0\leq j\leq k \end{array}}E\left[f\left(\text{diag}\left(i_{1,\;k}I_{m_{1}},\;i_{2,\;k}I_{m_{2}}\right),\;\cdots \;,\;\text{diag}\left(i_{1,\;0}I_{m_{1}},\;i_{2,\;0}I_{m_{2}}\right)\right)\right]\\ & \;\;\;\times P\left\{\gamma_{1,\;k}=i_{1,\;k},\;\cdots \;,\;\gamma_{1,\;0}=i_{1,\;0}\right\}P\left\{\;\gamma_{2,\;k}=i_{2,\;k},\;\cdots \;,\;\gamma_{2,\;0}=i_{2,\;0}\right\} \end{array}$$

(28) $$\begin{array}{cc} & \overset{\left(c\right)}{=}\;\sum_{\begin{array}{c} i_{1,\;j},i_{2,\;j}\in \left\{0,\;1\right\},\\ 0\leq j\leq k \end{array}}E\left[f\left(\text{diag}\left(i_{1,\;k}I_{m_{1}},\;i_{2,\;k}I_{m_{2}}\right),\;\cdots \;,\;\text{diag}\left(i_{1,\;0}I_{m_{1}},\;i_{2,\;0}I_{m_{2}}\right)\right)\right]\\ & \;\;\;\times P\left\{\gamma_{1,\;0}=i_{1,\;k},\;\cdots \;,\;\gamma_{1,\;k}=i_{1,\;0}\right\}P\left\{\;\gamma_{2,\;0}=i_{2,\;k},\;\cdots \;,\;\gamma_{2,\;k}=i_{2,\;0}\right\}\\ & =E\left[f\left(M_{0},\;\cdots \;,\;M_{k}\right)\right]\overset{\left(d\right)}{=}\;E\left[f\left(M_{1},\;\cdots \;,\;M_{k+1}\right)\right] \end{array}$$

Here, (a) is due to the definition of $M_{i}$. In view of the fact that $\left\{\gamma_{1,\;k}\right\}$ and $\left\{\gamma_{2,\;k}\right\}\;$ are dependent, we obtain (b). Combining Equations (4) and (27), we can verify for $l\in \left\{1,\;2\right\}$:

(29) $$\begin{array}{cc} P & \left\{\gamma_{l,\;k}=i_{l,\;k},\;\cdots \;,\;\gamma_{l,\;0}=i_{l,\;0}\right\}\\ & =\prod_{j=0}^{k-1}P\left\{\gamma_{l,\;j+1}=i_{l,\;j+1}\left|\gamma_{l,\;j}=i_{l,\;j}\right.\right\}P\left\{\gamma_{l,\;0}=i_{l,\;0}\right\}\\ & =\prod_{j=0}^{k-1}P\left\{\gamma_{l,\;j}=i_{l,\;j+1}\left|\gamma_{l,\;j+1}=i_{l,\;j}\right.\right\}P\left\{\gamma_{l,\;k}=i_{l,\;0}\right\}\\ & =P\left\{\gamma_{l,\;0}=i_{l,\;k},\;\cdots \;,\;\gamma_{l,\;k}=i_{l,\;0}\right\} \end{array}$$

which implies (c). The last equality (d) is derived by noting the assumption that ${\left\{\gamma_{i,\;k}\right\}}_{k\in N}$ starts at its stationary distribution. From Equation (28), we conclude that:

(30) $$E\left[\Lambda_{k}\right]=E\left[\Xi_{k}\right]$$

By Assumption 1, it yields that there exists a positive number ${\tilde{\beta }}_{1}$, such that:

(31) $$\sum_{i=1}^{\infty }A^{-iH}C^{H}CA^{-i}\leq {\tilde{\beta }}_{1}I_{n}$$

Thus, it follows from the above analysis:

(32) $$\begin{array}{cc} \Xi_{k} & \geq \sum_{i=1}^{k+1}A^{-iH}C^{H}M_{i}CA^{-i}+{\tilde{\beta }}_{1}^{-1}A^{-\left(k+1\right)H}\sum_{i=1}^{\infty }A^{-iH}C^{H}CA^{-i}A^{-\left(k+1\right)}\\ & =\sum_{i=1}^{k+1}A^{-iH}C^{H}M_{i}CA^{-i}+{\tilde{\beta }}_{1}^{-1}\sum_{i=k+2}^{\infty }A^{-iH}C^{H}CA^{-i}\\ & \geq \sum_{i=1}^{k+1}A^{-iH}C^{H}M_{i}CA^{-i}+{\tilde{\beta }}_{1}^{-1}\sum_{i=k+2}^{\infty }A^{-iH}C^{H}M_{i}CA^{-i}\\ & \geq \min\left(1,\;{\tilde{\beta }}_{1}^{-1}\right)\Gamma \end{array}$$

Together with Equation (30), we can prove:

(33) $$E\left[\Lambda_{k}^{-1}\right]\leq \max\left(1,\;{\tilde{\beta }}_{1}\right)E\left[\Gamma^{-1}\right]$$

By letting $\beta_{2}=\beta_{1}\max\left(1,\;{\tilde{\beta }}_{1}\right)$ and considering Lemma 3, we complete the proof.

Proof of the right-hand side: In view of Equation (23), it is obvious:

(34) $$\Xi_{k}\leq \sum_{i=1}^{\infty }A^{-iH}C^{H}M_{i}CA^{-i}+A^{-\left(k+1\right)H}A^{-\left(k+1\right)}$$

Thus, one has:

(35) $$\begin{array}{cc} & \underset{k\in N}{\sup}\;E\left[\Lambda_{k}^{-1}\right]=\underset{k\in N}{\sup}\;E\left[\Xi_{k}^{-1}\right]\\ & \;\;\;\geq \underset{k\in N}{\sup}\;E\left[{\left(\sum_{i=1}^{\infty }A^{-iH}C^{H}M_{i}CA^{-i}+A^{-\left(k+1\right)H}A^{-\left(k+1\right)}\right)}^{-1}\right]\\ & \;\;\;\overset{\left(e\right)}{=}\;\lim_{k\to \infty }\;E\left[{\left(\sum_{i=1}^{\infty }A^{-iH}C^{H}M_{i}CA^{-i}+A^{-\left(k+1\right)H}A^{-\left(k+1\right)}\right)}^{-1}\right] \end{array}$$

It is a simple matter to show that the right-hand side of Equation (35) is monotonically increasing with respect to *k*, which proves (e). Now, considering the monotone convergence theorem, one obtains:

(36) $$\begin{array}{cc} & \lim_{k\to \infty }\;E\left[{\left(\sum_{i=1}^{\infty }A^{-iH}C^{H}M_{i}CA^{-i}+A^{-\left(k+1\right)H}A^{-\left(k+1\right)}\right)}^{-1}\right]\\ & \;\;\;=E\left[\lim_{k\to \infty }\;{\left(\sum_{i=1}^{\infty }A^{-iH}C^{H}M_{i}CA^{-i}+A^{-\left(k+1\right)H}A^{-\left(k+1\right)}\right)}^{-1}\right]=E\left[\Gamma^{-1}\right] \end{array}$$

Combining Equations (35) and (36), one can easily show:

(37) $$\underset{k\in N}{\sup}\;E\left[\Lambda_{k}^{-1}\right]\leq E\left[\Gamma^{-1}\right]$$

Then, it follows from Lemma 3 that:

(38) $$\underset{k\in N}{\sup}\;E\left[P_{k}\right]\geq \alpha_{1}\underset{k\in N}{\sup}\;E\left[\Lambda_{k}^{-1}\right]\geq \alpha_{1}E\left[\Gamma^{-1}\right]$$

Let $\alpha_{2}=\alpha_{1}$, which completes the proof. ☐

We are now in the position to establish the relationship between the stability of error covariance matrix $P_{k}$ and the transition probabilities of the Markovian packet loss processes. Compared to the above theorem, the following result is restricted on non-degenerate systems. Hence, in the remaining part of the paper, we assume that:

**Assumption 4.**  The system $\left(C,\;A\right)$ is non-degenerate.

**Lemma 5.**  *Assume system $\left(C,\;A\right)$ satisfies Assumptions 1, 2 and 4. Then, there exists strictly positive constant number $\beta_{3}$, such that:*

(39) $$\lim_{\Delta_{1},\;\cdots \;,\;\Delta_{n}\to \infty }\;\frac{{\left(\sum_{j=1}^{n}A^{-k_{j}H}C^{H}CA^{-k_{j}}\right)}^{-1}}{\prod_{j=1}^{n}{\left|\lambda_{j}\right|}^{2\Delta_{j}}}\leq \beta_{3}I_{n},$$

*where $\lambda_{j}$$\left(1\leq j\leq n\right)$ is the eigenvalues of A, which satisfy $\left|\lambda_{1}\right|\geq \left|\lambda_{2}\right|\geq \cdots \geq \left|\lambda_{n}\right|$, $k_{1}<k_{2}<\cdots <k_{n}\in N$, $\Delta_{1}=k_{1}$, $\Delta_{j}=k_{j}-k_{j-1}$ for all $j\in \left\{2,\;\cdots \;,\;n\right\}$.*

**Theorem 6.**  Assume System (1) and (3) is a non-degenerate system, which satisfies Assumptions 1–3, and the packet loss process is described by a time-homogeneous Markov chain with transition probability matrix (8). Then, a necessary and sufficient condition for $\sup_{k\in N}E\left[P_{k}\right]<\infty$ is $\rho {\left(A\right)}^{2}\left(1-q_{1}\right)\left(1-q_{2}\right)<1$.

**Proof.**  Sufficiency: Based on Lemma 5, it suffices to show that there exists a sufficient, large positive constant number Δ, such that for all $\Delta_{j}>\Delta$, the following inequality holds:

(40) $${\left(\sum_{j=1}^{n}A^{-k_{j}H}C^{H}CA^{-k_{j}}\right)}^{-1}\leq \beta_{3}\prod_{j=1}^{n}{\left|\lambda_{j}\right|}^{2\Delta_{j}}I_{n}$$

We let $\tilde{C}=\min\left(C_{1}^{H}C_{1},\;C_{2}^{H}C_{2}\;\right)$ and select $k_{j}$ as follows:

(41) $$\begin{array}{cc} k_{1} & =\inf\left\{i>\Delta \left|S_{i}=\varsigma_{R}\right.\right\},\\ k_{j} & =\inf\left\{i>\Delta +k_{j-1}\left|S_{i}=\varsigma_{R}\right.\right\},\;j\in \left\{2,\;\cdots \;,\;n\right\} \end{array}$$

Obviously, it follows from Equations (24) and (40):

(42) $$\Gamma^{-1}\leq {\left(\sum_{j=1}^{n}A^{-k_{j}H}{\tilde{C}}^{H}\tilde{C}A^{-k_{j}}\right)}^{-1}\leq \beta_{3}\prod_{j=1}^{n}{\left|\lambda_{j}\right|}^{2\Delta_{j}}I_{n}$$

Thus, one can conclude that $E\left[\prod {}_{j=1}^{n}{\left|\lambda_{j}\right|}^{2\Delta_{j}}\right]<\infty$ implies $E\left[\Gamma^{-1}\right]<\infty$.

According to the similar idea in [9], we introduce stopping times as follows:

(43) $$\begin{array}{cc} d_{0} & =0,\\ d_{1} & =\inf\left\{i\geq 1\left|S_{i}=\varsigma_{R}\right.\right\},\\ d_{j} & =\inf\left\{i>d_{j-1}\left|S_{i}=\varsigma_{R}\right.\right\},\;j\in \left\{2,\;\cdots \;,\;n\right\} \end{array}$$

Moreover, sojourn time $\tau_{j}$, which is used to represent the time duration between two successive packet received times, is defined as:

(44) $$\tau_{j}=d_{j}-d_{j-1}$$

With regard to the definitions of $\Delta_{j}$ and $\tau_{j}$, one can find a sufficient large positive constant number *ε*, such that:

(45) $$E^{\varsigma_{R}}\left[\prod_{j=1}^{n}{\left|\lambda_{j}\right|}^{2\Delta_{j}}\right]\leq E^{\varsigma_{R}}\left[\prod_{j=1}^{n}{\left|\lambda_{j}\right|}^{2\left(\tau_{j}+\varepsilon \right)}\right]\overset{\left(f\right)}{=}\;E^{\varsigma_{R}}\left[\prod_{j=1}^{n}{\left|\lambda_{j}\right|}^{2\left(\tau_{1}+\varepsilon \right)}\right]<\infty$$

where (f) is due to Lemma 1 in [12], and the last inequality follows from the assumption $\rho {\left(A\right)}^{2}\left(1-q_{1}\right)\left(1-q_{2}\right)<1$.

Together with Equation (42), we now have $\sup_{k\in N}E^{\varsigma_{R}}\left[P_{k}\right]<\infty$. The other case with $S_{0}=\varsigma_{L}$ may be treated in the same manner. Finally, considering Lemma 1, we complete the proof.

Necessity: It has been verified in Theorem 2. ☐

**Corollary 7.**  *Consider the same situation as in Theorem 6, except for the number of sensors. We assume that the system is set with a distributed array of sensors (one or more), and each sensor offers partial measurements through a single channel. Then, a necessary and sufficient condition for $\sup_{k\in N}E\left[P_{k}\right]<\infty$ is:*

(46) $$\rho {\left(A\right)}^{2}\left(1-q_{1}\right)\left(1-q_{2}\right)\cdots \left(1-q_{l}\right)<1$$

*where l is the number of sensors and satisfies 1≤l≤m.*

**Proof.**  This corollary can be proven along the same line of the proof in Theorem 6. ☐

So far, we have obtained the necessary and sufficient stability condition for non-degenerate systems with all eigenvalues unstable. In the following, we generalize our result to systems with stable or marginally-unstable eigenvalues by recalling [9].

**Lemma 8.**  *Consider that System (1) and (3) satisfies Assumptions 2–3, and assume that matrix A is a diagonal matrix with the form of $A=diag\left(A_{1},\;A_{2},\;A_{3}\right)$, where $A_{1}$, $A_{2}$ and $A_{3}$ are the unstable, marginally unstable and stable parts, respectively. If matrix $C=\left[C_{r1},\;C_{r2},\;C_{r3}\right]$, then the critical value of the system can be bounded as:*

(47) $$\eta_{c}\left(A,\;C\right)\leq \lim_{\alpha \to 1^{+}}\;\eta_{c}\left(diag\left(\alpha A_{1},\;\alpha A_{2}\right),\;\left[C_{r1},\;C_{r2}\right]\right)$$

*where $C_{ri}$ is of appropriate dimensions and the critical value $\eta_{c}$ can be explained as follows. If the recovery rate $\eta >\eta_{c}$, then inequality $\sup_{k\in N}E\left[P_{k}\right]<\infty$ holds for all initial conditions. Otherwise, $E\left[P_{k}\right]$ is unbounded for some initial conditions.*

## 4. Discussion on Different Stability Criteria

As is well known, packet losses are inevitable due to unreliable communication channels under a network environment. Thus, compared to traditional filtering methods, estimation schemes for networked systems are required to deal with missing data. In most of the existing stability results for filtering under network environments, packet loss processes are modeled as an i.i.d. Bernoulli process or Markov chain. In this section, we compare the proposed results to existing related ones, which are under i.i.d. Bernoulli or Markov assumptions.

### 4.1. Comparison with Stability Results under i.i.d. Packet Losses

Under an i.i.d. Bernoulli packet loss assumption, the problem of filtering stability has attracted increased interest. Although technically challenging, it is possible to give an explicit characterization of the stability criteria for systems with certain restrictive conditions, e.g., invertibility on the observation matrix [17,19] or observable subspace [22], non-degeneracy on the pair $\left(C,\;A\right)$ [9].

Note that an i.i.d. process can be treated as a special case of the Markov chain. By letting the failure rate *μ* and recovery rate *η* in the transition probability matrix (8) satisfy μ=1−η, the Markov chain is reduced to an i.i.d. Bernoulli process with the arrival probability θ=η. Thus, the results given by Theorem 7 in [9] are recovered. In particular, for the case that part or all of the observation measurements are lost in an i.i.d. Bernoulli fashion, [19] proposes the following stability result:

(48) $$\rho {\left(A\right)}^{2}\left(1-\theta_{1}\right)\left(1-\theta_{2}\right)<1$$

where $\theta_{1}$ and $\theta_{2}$ represent packet arrival percentages of two different channels. We would like to point out that the above result can also be interpreted as a special case of our stability criterion in Theorem 6. Specifically, $\theta_{1}$ and $\theta_{2}$ correspond to $q_{1}$ and $q_{2}$, respectively, and the failure rate $p_{i}=1-q_{i}$, $i\in \left\{1,\;2\right\}$.

### 4.2. Comparison with Existing Stability Results under Markovian Packet Losses

To capture the temporal correlation of communication channels, we specialize the channel model as a Markov chain in this subsection. In contrast to the i.i.d. Bernoulli model, the approach by using the modified Riccati recursion is no longer feasible for Markovian packet losses, which makes the analysis for filtering stability more challenging.

In most of the existing results concerning the stability analysis of Kalman filtering, the single sensor case is assumed; while in practical network-based systems, various sensors and filter are usually at different physical locations, and thus, the single sensor assumption may not hold. On the basis of the above analysis, [20] analyzes the stability of the Kalman filter with partial packet losses by using Riccati recursion. However, the results in [20] are conservative, since a more restrictive condition that *C* is invertible is imposed on the system $\left(C,\;A\right)$. Obviously, as a special case of one-step observability, the above invertible condition requires that the matrix *C* must have *n* rows, which implies $y_{k}$ is at least a *n* dimensional vector. In contrast, under our non-degenerate assumption, *C* can only have *d* rows, where *d* is the dimension of the largest equi-block of the system. It is worth pointing out that *d* is usually smaller than *n* in the practical application. In addition, we give the necessary and sufficient condition for the stability of the mean estimation error covariance matrix, rather than only the sufficient condition as in [20].

## 5. Numerical Examples

In this section, for the purpose of illustrating the results from the previous sections, we present some simple examples.

**Example 1.**  *Consider a second order diagonal system with parameters expressed by:*

(49) $$A=diag\left(2.5,\;1.5\right),\;C=I_{2}$$

**Figure 3:**
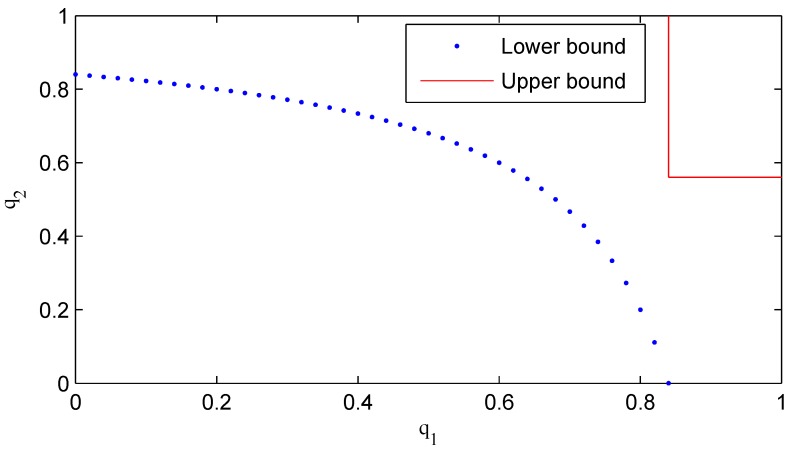
Stable and unstable regions.

Our aim is to compare the stable and unstable regions determined by our stability criteria and other existing stability results. For this system, [19] proposes that one needs $q_{1}>1-2.5^{-2}=0.84$ and $q_{2}>1-1.5^{-2}=0.56$ (upper bound) to guarantee the stability of the error covariance matrix; furthermore, one can conclude that the Kalman filter is unstable if the recovery rate pair $\left(q_{1},\;q_{2}\right)$ satisfies $\left(1-q_{1}\right)\left(1-q_{2}\right)>2.5^{-2}=0.16$ (lower bound). As shown in Figure 3, the region above the upper bound (red line) is stable, while the region below the lower bound (blue dotted line) is unstable. However, it can be seen from Figure 3 that the stability of the region between the upper and lower bounds cannot be determined with the results obtained by [19]. Recalling the stability result in [20], it is easy to check that the filter can achieve stability if only $\left(q_{1},\;q_{2}\right)$ falls above the lower bound; while the stability of the region below the lower bound cannot be determined since [20] only gives the sufficient condition for stability. We give the necessary and sufficient condition for stability in this paper. For this model, by Theorem 6, we can show that the region above the lower bound is stable; otherwise, it is unstable.

**Example 2.**  *Let a higher-order system be expressed by:*

(50) $$\begin{array}{cc} A & =diag\left(1.5,\;1.3,\;-1.3\right),\;C_{1}=\left[1\;0\;1\right],\;C_{2}=\left[1\;1\;0\right],\\ C & =\left[C_{1};\;C_{2}\right],\;Q=0.2I_{3},\;R=0.2I_{2} \end{array}$$

**Figure 4:**
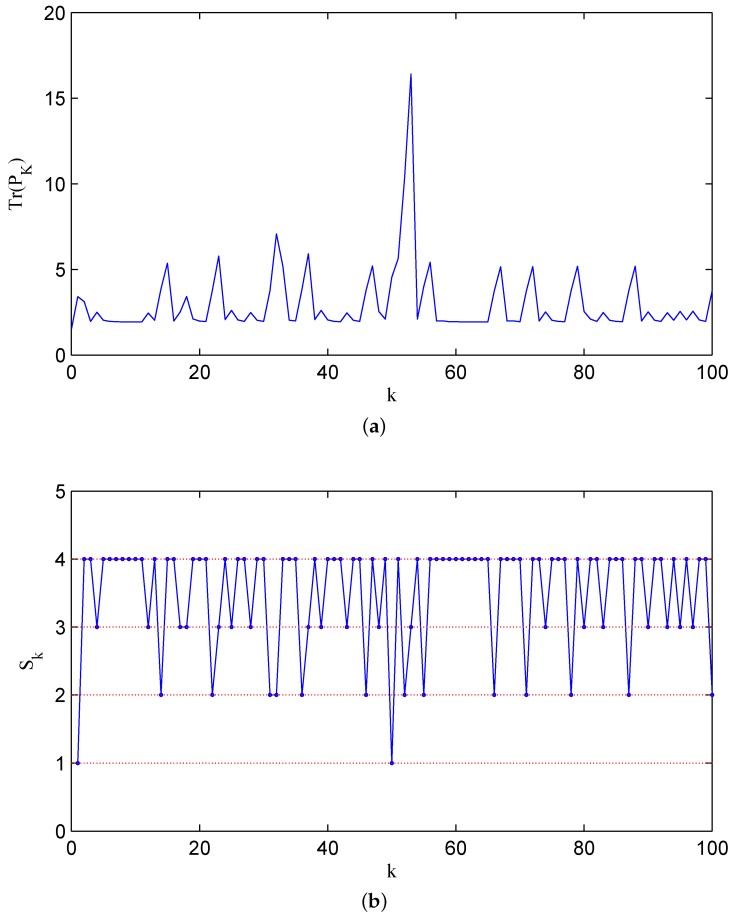
The error covariance matrix $P_{k}$ and channel state $S_{k}$ with $\left(q_{1},\;q_{2}\right)=\left(0.8,\;0.9\right)$: (**a**) The error covariance; (**b**) the associated channel state.

**Figure 5:**
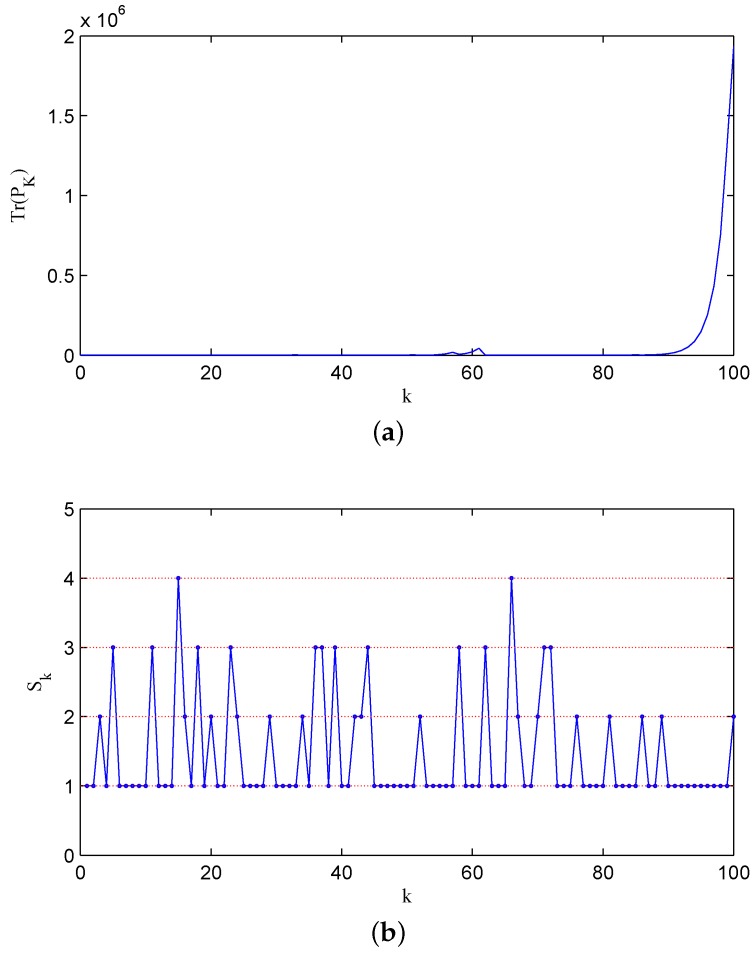
The error covariance matrix $P_{k}$ and channel state $S_{k}$ with $\left(q_{1},\;q_{2}\right)=\left(0.2,\;0.2\right)$: (**a**) The error covariance; (**b**) the associated channel state.

In order to guarantee stability, the recovery rate pair $\left(q_{1},\;q_{2}\right)$ should satisfy $\left(1-q_{1}\right)\left(1-q_{2}\right)<1.5^{-2}=0.44$ by Theorem 6. It is easy to check that the parameter pair $\left(q_{1},\;q_{2}\right)=\left(0.8,\;0.9\right)$ ensures the stability for the error covariance matrix of the filter. Figure 4a shows the change of the error covariance along that sample path, and in Figure 4b, the associated state of two channels jumping among $\varsigma_{00}$, $\varsigma_{01}$, $\varsigma_{10}$ and $\varsigma_{11}$ is displayed, where for the sake of simplicity, we note the states $\varsigma_{00}$, $\varsigma_{01}$, $\varsigma_{10}$ and $\varsigma_{11}$ as numbers 1, 2, 3 and 4, respectively. For comparison, we display a sample path with $\left(q_{1},\;q_{2}\right)=\left(0.2,\;0.2\right)$ in Figure 5a, and the associated channel state is shown in Figure 5b. The above two figures illustrate that with a lower recovery rate pair, the error covariance has more chances to diverge.

**Figure 6:**
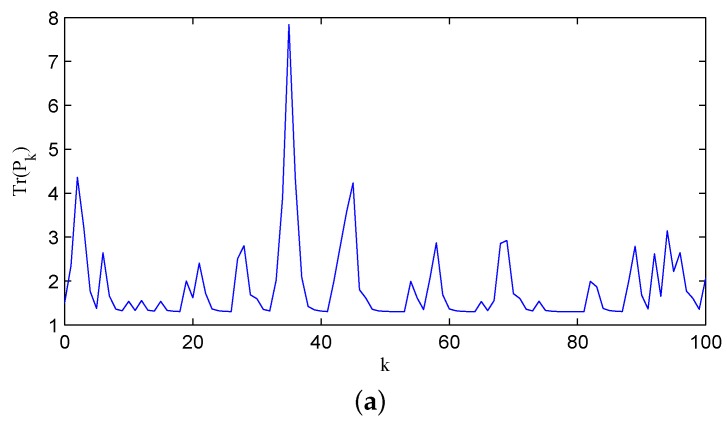

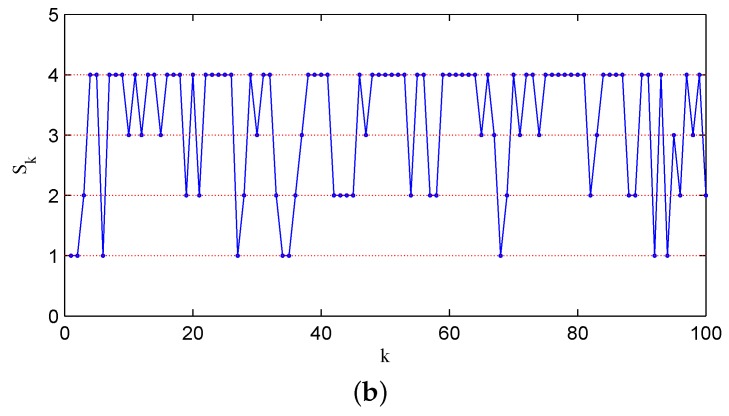
The error covariance matrix $P_{k}$ and channel state $S_{k}$ with $\left(q_{1},\;q_{2}\right)=\left(0.6,\;0.8\right)$: (**a**) The error covariance; (**b**) the associated channel state.

**Example 3.**  The results in Theorem 6 can be extended to the system with marginally unstable or/and stable eigenvalues by Lemma 8. Consider an example system specified by $A=diag\left(1.5,\;1,\;-0.5\right)$. The other parameters are chosen the same as those in Example 2. It is easy to check that $\left(1-q_{1}\right)\left(1-q_{2}\right)<0.44$ is sufficient to guarantee the stability of the error covariance matrix. Figure 6a shows a typical sample path with the recovery rate pair $\left(q_{1},\;q_{2}\right)=\left(0.6,\;0.8\right)$, while Figure 6b displays the associated channel state.

## 6. Conclusions

In this paper, we consider the problem of stability analysis for Kalman filtering under the multi-sensor environment. A necessary and sufficient condition is derived for ensuring the stability of the filter with partial Markovian packet losses. This condition is more general than the existing ones, since it only requires the system to be non-degenerate instead of one-step observable. Our results can recover related results in the literature, such as the case that the loss and non-loss channel states are described as i.i.d. process and the scenario where only one sensor is set. For future work, it is of interest to study the stability conditions for distributed filtering systems with multiple relevant communication channels.
